# Differential regulation of RNA polymerase III genes during liver regeneration

**DOI:** 10.1093/nar/gky1282

**Published:** 2018-12-29

**Authors:** Meghdad Yeganeh, Viviane Praz, Cristian Carmeli, Dominic Villeneuve, Leonor Rib, Nicolas Guex, Winship Herr, Mauro Delorenzi, Nouria Hernandez, the CycliX consortium, Nouria Hernandez, Mauro Delorenzi, Bart Deplancke, Béatrice Desvergne, Nicolas Guex, Winship Herr, Felix Naef, Jacques Rougemont, Ueli Schibler, Teemu Andersin, Pascal Cousin, Federica Gilardi, Pascal Gos, Fabienne Lammers, Maykel Lopes, François Mange, Shilpi Minocha, Sunil Raghav, Dominic Villeneuve, Roberto Fabbretti, Volker Vlegel, Ioannis Xenarios, Eugenia Migliavacca, Viviane Praz, Fabrice David, Yohan Jarosz, Dmitry Kuznetsov, Robin Liechti, Olivier Martin, Julien Delafontaine, Julia Cajan, Cristian Carmeli, Kyle Gustafson, Irina Krier, Marion Leleu, Nacho Molina, Aurélien Naldi, Leonor Rib, Jonathan Sobel, Laura Symul, Gergana Bounova, Philippe Jacquet

**Affiliations:** 1 Center for Integrative Genomics, Faculty of Biology and Medicine, University of Lausanne, 1015 Lausanne, Switzerland; 2 SIB Swiss Institute of Bioinformatics, 1015 Lausanne, Switzerland; 3 Bioinformatics Core Facility, SIB Swiss Institute of Bioinformatics, 1015 Lausanne, Switzerland; 4 Department of Fundamental Oncology and the Ludwig Center for Cancer research, Faculty of Biology and Medicine, University of Lausanne, 1015 Lausanne, Switzerland

## Abstract

Mouse liver regeneration after partial hepatectomy involves cells in the remaining tissue synchronously entering the cell division cycle. We have used this system and H3K4me3, Pol II and Pol III profiling to characterize adaptations in Pol III transcription. Our results broadly define a class of genes close to H3K4me3 and Pol II peaks, whose Pol III occupancy is high and stable, and another class, distant from Pol II peaks, whose Pol III occupancy strongly increases after partial hepatectomy. Pol III regulation in the liver thus entails both highly expressed housekeeping genes and genes whose expression can adapt to increased demand.

## INTRODUCTION

Compared to RNA polymerase (Pol) II promoters, Pol III promoters are quite simple with just three main types of structures. Type 1 promoters occur only in the 5S genes, type 2, by far the most abundant, are present in tRNA genes, most SINEs, and some other genes, and type 3 are present in less than fifteen annotated genes in both the human and mouse genomes (1,2). Despite this relative uniformity, different annotated Pol III genes have very different levels of Pol III occupancy, which correlate with different levels of transcriptional activity (3–5). In fact, one of the surprises of early genomic studies was the discovery that some 40–50% of annotated Pol III genes are not occupied by Pol III and transcriptionally silent, an observation that is only partially explained by poor promoter sequences (2–4,6–9).

Expressed Pol III loci differ from silent ones by the nearby presence of histone marks such as H3K4me3 (2,6–8,10) typical of chromatin regions that are or have been actively transcribed by Pol II ((11) and references therein). Moreover, active Pol III loci tend to reside close to Pol II TSSs and to peaks of Pol II occupancy, which suggests that transcription of nearby Pol II and Pol III genes is somehow co-regulated (2,5–8,10). Pol III transcription is indeed tightly regulated, allowing the cell to adapt to changing needs in biosynthetic capacity resulting from, for example, cell growth and proliferation. Furthermore, overexpression of Pol III genes is observed in many transformed cells (12–17). Similarly, genome-wide Pol III occupancy comparisons of mouse hepatocarcinoma cells with normal mouse liver cells (18), or of precursors with induced pluripotent cells and human H1 ES cells (10), all point to higher Pol III transcription in dividing as compared to differentiated cells.

Genome-wide Pol III occupancy and transcription have been studied in only a few dynamic systems, and very rarely in a normal tissue. Studies comparing Pol III occupancy in human serum-starved versus serum-replete IMR90Tert cells (4), in the mouse liver at different times during the circadian cycle (19), in mouse liver and brain at different stages of development (20), or in THP-1 cells and THP-1-derived macrophages by PMA treatment (5) have all emphasized that different Pol III genes respond differently to changing cellular conditions. In the last case, concerted down-regulation of certain tRNA genes in clusters and contact domains was observed.

Here, we have taken advantage of the synchronous hepatocyte proliferation occurring after partial hepatectomy (PH) to examine, in a normal tissue, the dynamics of Pol III occupancy upon transition from a resting G0 state to a proliferating state. We find two classes of active Pol III genes, one class with high and relatively static Pol III occupancy, often characterized by proximity to Pol II TSSs and Pol II peaks, and a second class with much lower Pol III occupancy, devoid of nearby Pol II peaks, but highly dynamic. The resulting picture is one where a network of Pol III genes, often located close to Pol II TSSs, ensures steady production of essential Pol III RNA products in the differentiated tissue, whereas another, expressed at low levels in the differentiated tissue, ensures the increased synthesis of Pol III products needed in preparation for cell division.

## MATERIALS AND METHODS

### Animals, partial hepatectomies, and chromatin immunoprecipitations (ChIP)

C57/BL6 12–14-week-old male mice were housed under a 12 h light/12 h dark cycle regimen for two weeks with food available during the night. Two-third partial hepatectomies were performed as described (21–23). Three pools of three mice were processed in one batch between ZT01.5 and ZT02.5, with three mice operated every 20 minutes. The livers of the three mice were pooled for each timepoint. ChIPs were performed as described (24). The following antibodies were used: anti-RPC4 (CS681) (2), anti-H3K4me3 (Abcam, ab8580) and anti-RPB2 (Santa Cruz Biotechnology, sc-673-18). It should be noted that the anti-H3K4me3 antibody used scored as the best ENCODE-validated anti-H3K4me3 antibody but is 60–66% specific for H3K4me3, with crossreaction to H3K4me2 and very weak crossreaction with H3K4me1 (25).

### Ultra-high-throughput sequencing and tag alignment

Ten nanogram of immunoprecipitated chromatin was used to prepare sequencing libraries with the Diagenode MicroPlex Library Preparation kit (catalog no C05010011) as specified by the manufacturer, with a total of 14 amplification cycles. One or several bar-coded sequencing libraries were then loaded onto one lane of a HiSeq 2000 flow cell and paired-end sequenced at 50 or 100 cycles. For each condition, we sequenced both input chromatin and the corresponding ChIP samples.

The first fifty nucleotides of each sequence were mapped onto the UCSC mouse genome version NCBI37/mm9 via the eland_extended mode of ELAND v2e in the Illumina CASSAVA pipeline v1.8.2. We first retrieved fragments with unique matches at both ends. The tags with multiple matches on the mouse genome were then aligned via fetchGWI with an allowed maximum of 500 matches per tag. When tag alignment revealed multiple possible fragments for a tag pair, all possible fragments were kept and given a weight corresponding to the normalized size probability as determined from the size probability distribution of the unique fragments. Only fragments sequenced once (non-redundant fragments) were considered in the analysis.

### Score computation

We first normalized the number of fragments aligned onto the genome in each sample relative to the median number of fragments aligned onto the genome across all samples (all time points). Scores were computed as the log_2_-ratio between counts in paired ChIPs and Input samples to which a pseudocount value of 16 was added. For RPC4 ChIP scores, the counts were assigned to a previously defined list of mouse annotated Pol III genes and Pol III occupied loci (see (18)), with each annotated RNA-coding region extended by 150 bp on each side. As shown in Supplementary Figure S1, the correlation between the RPC4 scores calculated from paired-end sequencing (this work) and single-end sequencing (18) was very high (*R* = 0.962). For H3K4me3 and RPB2, the regions considered extended 1000 bp upstream and downstream of the Pol III loci TSSs. One fragment was worth one count, and fractional counts were attributed in case of a partial overlap between a fragment and an extended locus.

To establish whether the score value at a particular locus was significantly higher than in other regions of the genome, the score was compared to the distribution of scores computed in non-overlapping bins (400 bp for RPC4 and 2000 bp for H3K4me3 and RPB2) over the entire genome. The threshold value was obtained applying a Bonferroni correction for the multiplicity of loci. The calculated scores for all samples are listed in Supplementary Table S1.

### Data quality

To monitor sample quality, we produced mean-difference scatter plots of mouse genome bin counts and Pol III loci (see (26)). Samples displaying well-differentiated RPC4 occupancy scores on Pol III loci compared to scores on the rest of the genome were considered of good quality. 134 tRNA genes out of 433 (31%) had scores below the cutoff in all replicates and all time points. Supplementary Figures S2A and B show the score reproducibility for biological replicates obtained at TP0, TP36, TP48, and TP60, for RPC4 and RPB2 occupancy, respectively. The scores from replicates are, in some cases, slightly shifted relative to the X = Y line, but the correlation coefficients are always larger than 0.9.

### Statistical modeling

To investigate dynamical changes of RPC4 scores we considered a general linear model with four levels (corresponding to the four time points, i.e. TP0, TP36, TP48 and TP60) and two replicates per level and per ChIP-seq. The model was estimated through empirical Bayes method implemented in the Limma package (27). That method allows a robust estimation of the variance ensuing from the variance across genes. For each contrast of interest (e.g. TP36 versus TP0) a *P*-value and a T statistic were computed for each locus. To take into account the multiplicity of null hypotheses, false discovery rates (FDR) were estimated from the *P*-values (28). In particular, we estimated the proportion of null *P*-values through a smoothing spline approach (29) to increase power. Statistical significance was called for genes showing a FDR <0.05.

### Gene expression analysis

Total RNA was extracted using miRNeasy mini kit (Qiagen) with on-column DNase I treatment. One microgram of RNA was used for cDNA synthesis with random hexamers and M-MLV Reverse Transcriptase (Promega). qPCRs were performed with appropriate primers (see Supplementary Table S2) and SensiFAST SYBR kit (Bioline) under the following conditions: an initial denaturing step (95°C 10′) followed by 40 cycles of incubations at 95°C for 15″, 58°C for 20″, and 72°C for 20″. qPCR reactions were performed in triplicate, with reactions lacking reverse transcriptase serving as negative controls. Melting curve analyses revealed single peaks for all reactions, and calculation of primers efficiencies using standard curves indicated comparably high efficiencies (from 86 to 102%) for the different primer sets. The relative expressions were analyzed with the 2^ΔΔCt^ method.

## RESULTS AND DISCUSSION

### Association of RPC4 occupancy with H3K4me3 and RPB2 occupancy at TP0

In our experiments, mice were kept in 12 h light/12 h dark cycle, with food available only during the night. PH was performed always at the very beginning of the day (ZT01.5-ZT02.5). We profiled H3K4me3, Pol II (RPB2 subunit), and Pol III (RPC4 subunit) occupancy in parallel at different time points after PH by chromatin immunoprecipitation followed by high throughput sequencing (ChIP-seq). The Pol II and H3K4me3 occupancy data have been used to study occupancy dynamics at Pol II genes (11). Here, we used the same data as well as the Pol III data to determine occupancy scores at Pol III genes, as described before (18) (Supplementary Table S1).

We first explored the relationship between RPC4 occupancy and presence of H3K4me3 and RPB2 peaks at TP0, before PH. To avoid confusing signals from overlapping peaks at closely spaced loci, we considered only ‘isolated’ loci, i.e., Pol III loci separated by at least 1.5 kb, unless otherwise mentioned (437 out of 646 loci, Supplementary Table S3, column D). We classified these loci into eight groups, i.e., loci with a peak for each of the three factors tested, with peaks for two of them, with a peak for only one of them, and with no peaks (Table 1). The Rn5S and the Rn4.5S genes, which are each tandemly repeated in the genome, displayed only RPC4 peaks, and both RPC4 and H3K4me3 peaks, respectively (Table 1 and Supplementary Table S3). We found SINEs in all groups, whereas nearly all ‘other’ Pol III genes, corresponding mostly to genes with type 3 promoters (see Supplementary Table S3, genes labeled ‘other’, for a list), had peaks for RPC4+H3K4me3+RPB2 (Table 1).

**Table 1.** Pol III loci grouped according to presence or absence of RPC4, H3K4me3 and RPB2 peaks

		Pol III	Pol III	Pol III	——-	Pol III	——-	——-	——-
		Pol II	Pol II	——-	Pol II	——-	Pol II	——-	——-
		H3K4me3	——-	H3K4me3	H3K4me3	——-	——-	H3K4me3	——-
tRNA genes (233)	No.	37/29	0/0	41/49	2/2	24/22	3/1	1/1	125/129
	%	15.9/12.4	0/0	17.6/21	0.9/0.9	10.3/9.4	1.3/0.4	0.4/0.4	53.6/55.4
SINEs (123)	No.	18/9	9/2	10/5	7/14	42/23	2/6	5/12	30/52
	%	14.6/7.3	7.3/1.6	8.1/4.1	5.7/11.4	34.1/18.7	1.6/4.9	4.1/9.8	24.4/42.3
Rn5s (51)	No.	0/0	0/0	0/0	0/0	40/40	0/0	0/0	11/11
	%	0/0	0/0	0/0	0/0	78.4/78.4	0/0	0/0	21.6/21.6
Rn4.5s (17)	No.	0/0	0/0	17/17	0/0	0/0	0/0	0/0	0/0
	%	0/0	0/0	100/100	0/0	0/0	0/0	0/0	0/0
Other Pol III genes (13)	No.	11/11	0/0	1/1	1/1	0/0	0/0	0/0	0/0
	%	84.6/84.6	0/0	7.7/7.7	7.7/7.7	0/0	0/0	0/0	0/0

The columns correspond to isolated loci grouped by presence or absence (as defined by a score above or below the cut-off) of Pol III, H3K4me3 or Pol II, at TP0. The lanes correspond to different types of Pol III loci. The first number in each group corresponds to RPC4 and RPB2 replicates 1, and H3K4me3 replicate 2, the second to RPC4, RPB2 and H3K4me3 replicates 2. For each group, the percentage of total number of genes in the corresponding gene type (shown in parenthesis in the first column) is indicated.

For tRNA genes, the largest group was the one with no peaks (Table 1). Considering just Pol III occupancy, ∼56% of isolated tRNA genes had no RPC4 peaks against ∼45% of all tRNA genes (see Supplementary Table S3), indicating that the proportion of silent tRNA genes is higher among isolated genes than among all tRNA genes. This is in line with the finding that in human cells, the median expression level for tRNA genes increases with the number of neighboring tRNA genes (5). The second and third largest groups of isolated tRNA genes were the ones with both RPC4+ H3K4me3 peaks, and with RPC4 + H3K4me3 + RPB2 peaks, respectively. The group with RPC4 peaks only comprised about 10% of isolated tRNA genes, and the latter proportion was similar for all tRNA genes (Table 1 and Supplementary Table S3).

**Figure 1.**
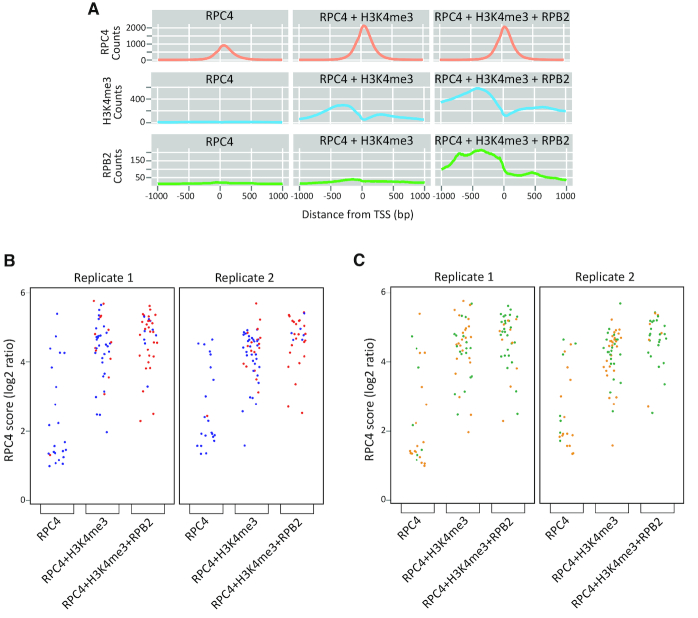
(**A**) Average tag density profiles for the factors indicated in the left, for the isolated tRNA gene groups (columns). The profiles were computed from replicate 2. (**B**) Scatterplots of RPC4 scores for isolated tRNA genes in replicate 1 and 2. The grouping into tRNA gene groups was done for each replicate independently (for H3K4me3, only replicate 2 was used). *P*-value < 2e–4 for the two comparisons in both replicates (see text), permutation based t test with 10000 permutations. Red and blue dots, genes with and without associated CpG islands, respectively. (**C**) As in B, but the orange and green dots indicate genes >2.65 kb and <2.65 kb, respectively, from TSS or poly A site of Pol II genes.

We then investigated whether the RPC4 occupancy scores differed across groups and types of genes. We focused on the groups with RPC4 peaks either i) alone or ii) with H3K4me3 peaks or iii) with both H3K4me3 and RPB2 peaks. The average RPC4 tag density profile and the individual gene RPC4 occupancy scores were lowest in the first group and much higher in the groups with H3K4me3 peaks or H3K4me3 + RPB2 peaks for isolated tRNA genes (Figures 1A and B), an effect not seen for isolated SINEs (Supplementary Figures S3A and B). RPC4 scores of tRNA genes with RPC4 + H3K4me3 peaks, or RPC4 + H3K4me3 + RPB2 peaks, were similar to each other but clearly higher than scores of tRNA genes with only RPC4 peaks (Figure 1B). The SINEs in the three groups had very similar scores (Supplementary Figure S3B). Comparing tRNA gene and SINE scores in each of the three groups revealed no significant difference for loci with only RPC4 (*P*-value = 0.1162 and 0.0126 for replicates 1 and 2) but much higher scores for tRNA genes with RPC4 + H3K4me3 peaks, or RPC4 + H3K4me3 + RPB2 peaks (*P*-value < 2e–4).

The average tag density RPC4 profiles revealed low peaks of RPC4 in RPC4 alone group (Figure 1A and Supplementary Figure S3A, see also Supplementary Figure S4A). H3K4me3 profiles for genes with RPC4+H3K4me3 peaks revealed a prominent H3K4me3 peak upstream of the TSS, and for tRNA genes a smaller one downstream, reflecting the aggregate of individual tRNA genes with only an upstream peak, only a downstream peak, or both (see Supplementary Figure S4B). In the case of tRNA genes and SINEs in the third group, the profiles of accumulated tags were very similar, with major upstream and minor downstream peaks (Figure 1A, Supplementary Figures S3A and S4C). This directionality, with the Pol II peak generally upstream of the Pol III peak, is consistent with previous observations in cultured human cells (6,7,10,30).

CpG islands (within 1 kb around the TSS) (red dots in Figure 1B and Supplementary Figure S3B, Supplementary Table S3, column F) were strongly associated with the presence of RPB2 peaks and depleted in tRNA genes or SINEs with RPC4 peaks only. Thus, at TP0, isolated SINEs tend to have low Pol III occupancy regardless of the presence of H3K4me3 or RPB2 peaks, whereas isolated tRNA genes can have high Pol III occupancy scores, which are generally associated with the presence of nearby H3K4me3 and RPB2 peaks.

The general proximity of active Pol III transcription units to H3K4me3 and Pol II peaks, and to Pol II TSSs, has been noted before (2,5–8,10,30), but the relationship among these Pol II peaks, H3K4me3 peaks, and Pol II TSSs is not clear. Separation of isolated Pol III genes into two groups, those within 2.65 kb of, and those removed by more than 2.65 kb from, a Pol II gene (TSS or poly A signal) (Supplementary Table S3, column D) revealed that the large majority (about 76% for tRNA genes and 89% for SINEs) of the loci with a RPB2 (and H3K4me3) peak were close to known Pol II genes (Figure 1C and Supplementary Figure S3C).

Visual examination of each of these <2.65 kb loci showed that in 26 out if 27 cases for tRNA genes, and in 13 out of 16 cases for SINEs, the RPB2 and H3K4me3 peaks overlapped a Pol II TSS (See Supplementary Figure S5A for examples), and the three remaining SINEs were located within Pol II transcription units. Pol II TSSs and tRNA genes were often divergent, consistent with previous findings (8) and explaining the major Pol II peak upstream of tRNA genes observed above (Figure 1A). Further, of the 10 isolated tRNA loci with an RPB2 peak >2.65 kb away from a Pol II TSS, half contained a CpG island (see Supplementary Figure S5B for examples with and without a CpG island). Thus, for most isolated tRNA genes and most SINEs with RPC4 + H3K4me3 + RPB2 peaks, the H3K4me3 and RPB2 peaks are explained by Pol II occupancy of a nearby Pol II transcription unit TSS or, for a few tRNA genes, of a nearby CpG island devoid of annotated TSSs. In HeLa cells, Oler *et al.* (8) observed a much more general presence of Pol II peaks upstream of Pol III-occupied tRNA genes, independent of the presence of Pol II TSSs. This may reflect a human-mouse cell difference or, more likely, a more spurious Pol II occupancy in the highly transformed HeLa cells as opposed to normal mouse liver cells.

In the RPC4 + H3K4me3 tRNA gene group close to Pol II loci (Figure 1C, green dots), the H3K4me3 peak was on a nearby Pol II TSS in only half the cases; in the other half, the tRNA gene was often surrounded by two H3K4me3 peaks with no associated annotated feature (see Supplementary Figure S5C for examples). In RPC4 + H3K4me3 tRNA gene group far from Pol II loci, there was generally a single major H3K4me3 peak on one side of the tRNA gene, in rare cases associated with a CpG island (Supplementary Figure S5D). These findings confirm the occurrence of H3K4me3 peaks close to active Pol III genes independent of the presence of Pol II peaks. Indeed, the 4.5S genes are a striking example of this configuration (See Table 1). This suggests that H3K4 trimethylation can occur at Pol III transcription units independently of the nearby presence of Pol II and Pol II transcription units. Such trimethylation might be brought about through transcription factors such as MYC, which can associate both with active Pol III genes (31), and with the WDR5 subunit of H3K4 methylase complexes (32).

Thus, at tRNA genes, high RPC4 occupancy scores are associated with proximity to either just H3K4me3 or H3K4me3 + RPB2 peaks, and in the latter case the H3K4me3 and RPB2 peaks are generally associated with Pol II TSSs. At SINEs, the RPC4 scores are similar in all groups, but where RPB2 and H3K4me3 peaks are present, these peaks are again mostly associated with Pol II TSSs or lie within Pol II transcription units.

### Changes in Pol III genome association after PH

**Figure 2.**
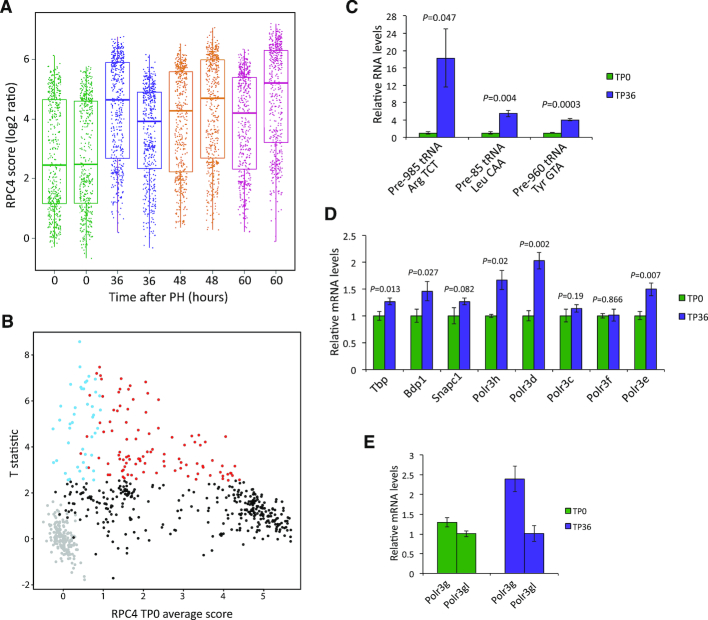
(**A**) Distribution of RPC4 scores for Pol III genes across time and replicates. Dots represent Pol III genes, and their distribution is summarized with their median (thicker horizontal bar), lower/upper quartile (box) and ±1.5 times the interquartile range (end of whiskers). Genes with an RPC4 occupancy score below the cutoff in all time points and replicates are not shown. (**B**) Plot of T statistics estimated for the TP0 to TP36 transition as a function of the average RPC4 score across replicates at TP0. A positive value of the T statistic indicates a score increase from TP0 to TP36. Red and blue dots, loci with an associated FDR<0.05, with blue dots indicating loci with an RPC4 score below cutoff at TP0. Black dots, loci with an associated FDR >0.05. Gray dots, loci with scores below the cutoff at both TP0 and TP36. (**C**) RT-qPCR quantification (normalized to Actb mRNA level) of indicated precursor tRNAs, relative to the values at TP0. Error bars represent ± SD. *n* = 3. The *P*-values were calculated using Student's *t*-test. (**D**) RT-qPCR quantification (normalized to Actb mRNA level) of the indicated mRNAs, relative to the values at TP0. Error bars and *P*-values as in C. (**E**) RT-qPCR quantification of *Polr3g* relative to *Polr3gl* mRNA. Error bars as in C.

The results above describe Pol III gene occupancy in the liver, an organ composed largely of differentiated cells in a G0 state. After PH, most of the remaining hepatocytes exit from the G0 state and progress though the cell division cycle. Under the conditions used here for PH, S phase was reached after about 36 h (time point (TP) 36), G2/M phase at 44–48 h (TP48), followed by a second, less synchronous round of division (23). We examined RPC4 occupancy scores after PH. In general, RPC4 occupancy at all Pol III loci (isolated or not) increased sharply from TP0 to TP36, and then stayed quite elevated at TP48 and TP60 (Figure 2A). We built a one-way ANOVA statistical model with samples from TP0, TP36, TP48 and TP60 and ran an estimation of the contrasts of interests with the empirical Bayes method (see Materials and Methods). We did not find statistical differences (score changes with an FDR < 0.05) between the samples at TP36, TP48 and TP60. There was, however, a significant difference between TP0 and the later time points. We focused on the TP0 to TP36 transition, corresponding to liver cells in G0 (TP0) and S phase (TP36).

To illustrate the RPC4 score changes from TP0 to TP36, we plotted the T statistic as a function of the average score at TP0 (Figure 2B; see Supplementary Table S4 for FDR and T statistic values). The RPC4 scores of most loci increased, but 143 loci showed significant changes (red and blue dots; FDR < 0.05) and corresponded to those with low to medium RPC4 scores at TP0; in fact, 44 loci with significant changes from TP0 to TP36 had an RPC4 score below cutoff at TP0 (blue dots). Thus, out of 433 tRNA genes, 295 were occupied by Pol III in at least one sample at TP0 or TP36. By comparison, 300 tRNA genes were found to be active in at least one stage of mouse liver development (20), among them the 295 found active here. This indicates that as suggested for human tRNA genes (4), a population of mouse tRNA genes is in a lasting repressed state.

In contrast to loci with low scores at TP0, most loci with high scores at TP0 did not change significantly. Interestingly, out of the 143 changing genes with significant changes, the most numerous ones (n=107), and the ones with the largest changes (representing 100% of the genes in the upper tertile) were tRNA genes. The remaining changing loci were SINEs (n=31), Rn5s (n=3), and other Pol III genes, namely Rny1 and Rpph1.

To determine whether increased Pol III occupancy reflected increased transcription, we measured levels of intron-containing tRNA precursors, which are unstable and, therefore, better reflect transcription activity than mature tRNAs. RT-qPCR revealed increases in precursor tRNA levels derived from the 985-tRNAArg-TCT, 85-tRNALeu-CAA, and 960-tRNATyr-GTA loci (Figure 2C), consistent with increased Pol III occupancy reflecting increased ongoing transcription (4,5).

The increase in Pol III transcription was accompanied by increased levels of mRNAs coding for several transcription factors and Pol III subunits including TBP, BDP1, RPC8 (*Polr3h* mRNA), RPC4 (*Polr3d* mRNA), and RPC5 (*Polr3e* mRNA) (Figure 2D), in line with the observation that upon transformation of cultured cells, the levels of some Pol III transcription factors and subunits increase (17). We also observed an increase in the ratio of *Polr3g* to *Polr3gl* mRNA levels (Figure 2E), consistent with the observation that the *Polr3gl* gene provides a basic protein level and the *Polr3g* gene allows adaptation to conditions when more protein is needed ((18) and references therein).

The observation that only a subset of tRNA genes displayed significantly increased RPC4 occupancy at TP36 as compared to TP0 prompted us to investigate how this increased occupancy affected tRNAs by isotypes and isoacceptors. As shown in Supplementary Figures S6A and S6B, the increase was general among tRNA isoacceptors and isotypes, suggesting a translation globally more active in dividing cells as compared to resting ones. We further wondered whether tRNA genes differentially occupied by Pol III at TP0 and TP36 were the same as the ones changing during development, and thus compared them with those changing between embryonic stage E15.5 and adult mouse liver (33). Of the 107 tRNA genes with changing scores from TP0 to TP36 and the 97 genes with changing scores from E15.5 to adult liver, 51 were common, clearly more than expected by chance (Supplementary Figure S7). Thus, there seems to be a pool of tRNA genes that are especially susceptible to differential regulation.

### Changes in Pol III occupancy after PH correlate weakly with changes in H3K4me3 but not with changes in RPB2 occupancy

**Figure 3.**
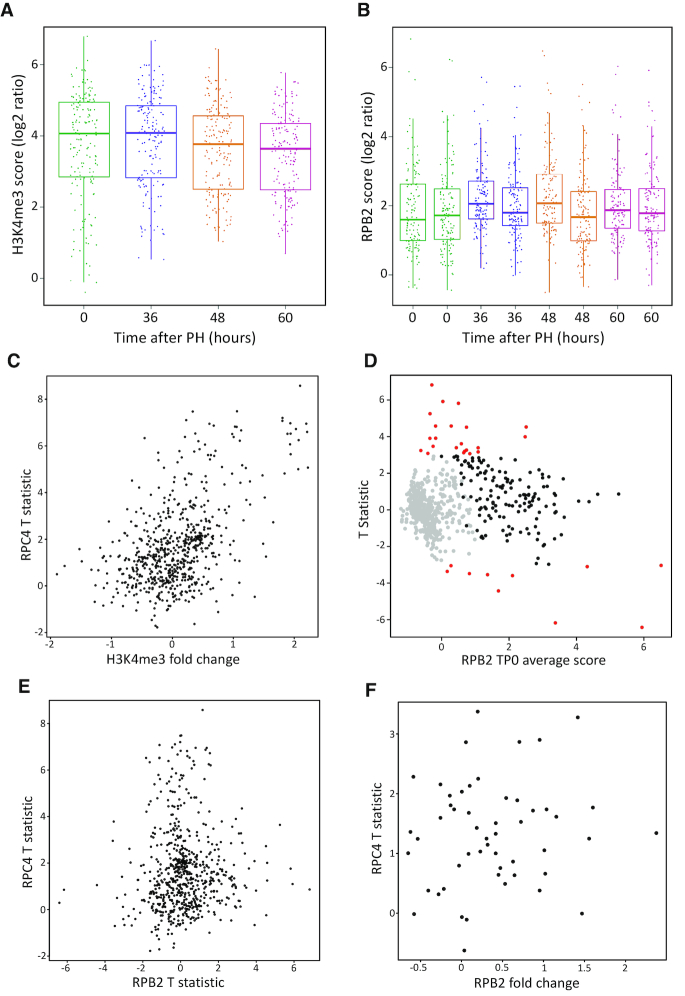
(**A**) Distribution of H3K4me3 scores around Pol III genes across time. Dots represent isolated Pol III loci whose H3K4me3 scores are above the cutoff. (**B**) Distribution of RPB2 scores around Pol III genes across time and replicates. Dots represent isolated Pol III loci whose RPB2 scores are above the cutoff. (**C**) Scatterplot of RPC4 scores T statistic and H3K4me3 fold change from TP0 to TP36. All Pol III loci are shown (*n* = 646). (**D**) Plot of RPB2 scores T statistic estimated for the transition TP0 to TP36 as a function of the RPB2 average score, across replicates, at TP0. RPB2 scores were computed in a region of ±1 kb around Pol III gene TSS. A positive value of the T statistic indicates an increase of score from TP0 to TP36. Red dots and black dots, genes with an associated FDR< and >0.05, respectively; grey dots, genes with RPB2 scores below cut-off. (**E**) Scatterplot of RPC4 and RPB2 scores T statistic, from TP0 to TP36. All Pol III loci are shown (*n* = 646). (**F**) Scatterplot of RPC4 T statistic and fold change of RPB2 occupancy at nearby Pol II TSS (±250 bp of the Pol II TSS). The Pol III loci considered (both isolated and not isolated) had RPC4 scores above the cutoff and were within 2.65 kb of a Pol II TSS with a corresponding RPB2 peak (*n* = 52).

As shown above and consistent with previous observations in cultured cell lines, hepatocytes respond to the need to divide by differential increased Pol III transcription at individual Pol III loci. Do these loci also display changes in nearby H3K4me3 or Pol II occupancy? Both H3K4me3 and RPB2 scores around Pol III loci were overall quite stable (Figures 3A and B). We observed a weak correlation (Pearson correlation coefficient = 0.51) between changes in Pol III and surrounding H3K4me3 peaks at all Pol III loci (Figure 3C, see also Supplementary Table S5), which was slightly higher for Pol III loci with RPC4 scores above cutoff in at least one sample at TP0 or TP36 (Pearson correlation coefficient = 0.56, data not shown). In contrast, whereas most Pol III occupancy changes were positive from TP0 to TP36, Pol II occupancy changes went in both directions (Figure 3D and Supplementary Table S4), and the correlation was very low (Figure 3E and Supplementary Table S5, Pearson correlation coefficient = 0.04). On the other hand, Pol III occupancy changes and Pol II occupancy changes occurring specifically at nearby Pol II TSSs (±250 bp) were generally positive for both Pol II and Pol III, consistent with the observations of Van Bortle *et al.* (5), who observed tRNA genes in domains to be down-regulated similarly to nearby protein-coding genes during differentiation (Supplementary Table S5). However, the Pearson correlation was again very low (0.12) (Figure 3F). Thus, locally (±1 kb), on a gene per gene basis, the dynamical changes are not quantitatively associated. This argues against a simple model in which Pol III genes are submitted to the same transcription regulation as nearby Pol II transcription units.

### Pol III occupancy dynamics after PH in different groups

**Figure 4.**
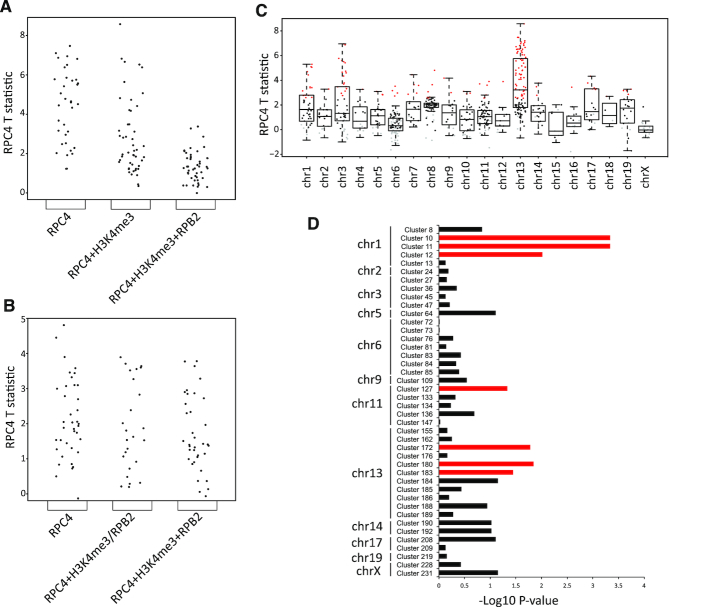
(**A**) Scatterplot of RPC4 T statistic (between TP0 and TP36) for the indicated groups of isolated tRNA genes (RPC4 peak only, *n* = 35; RPC4 + H3K4me3 peaks, *n* = 48); RPC4 + H3K4me3+RPB2 peaks (*n* = 46). There are significant differences between groups (see text for *P*-values). (**B**) Scatterplot of T statistic (between TP0 and TP36) for the indicated groups of isolated SINEs (RPC4 peak only, *n* = 41; RPC4+H3K4me3 peaks, *n* = 11; RPC4 + RPB2 peaks, *n* = 14; RPC4 + H3K4me3 + RPB2 peaks, *n* = 38). There was no significant difference between various groups (RPC4 only versus RPC4+H3K4me3/RPB2, *P*-value = 0.2417; RPC4 versus RPC4 + H3K4me3 + RPB2, *P*-value = 0.1018; RPC4 + H3K4me3/RPB2 versus RPC4 + H3K4me3 + RPB2, *P*-value = 0.9975). (**C**) RPC4 T statistic for TP0 to TP36 transition across different chromosomes. Red dots, loci with significant changes, grey dots, loci with RPC4 scores below cutoff. (**D**) Comparison of RPC4 occupancy fold change distribution between tRNA genes in clusters and outside clusters on the same chromosome. Red bars indicate clusters with KS test *P*-value <0.05.

We next wondered whether there might be differences in how Pol III occupancy scores change in Pol III genes with different associated peaks. Whereas for SINEs, there were no significant differences between the groups, for tRNA genes, T statistic scores were highest in the RPC4 peak only group (two-sided KS test *P*-value = 0.001439 for RPC4 > RPC4 + H3K4me3, and 3.073e–11 for RPC4 > RPC4 + H3K4me3 + RPB2), and slightly higher in the RPC4 + H3K4me3 peak group than in the RPC4 + H3K4me3 + RPB2 group (*P*-value = 0.0004566) (Figures 4A and B, see also Supplementary Table S6). We focused on the differences between the RPC4 peak only and the RPC4 + H3K4me3 + RPB2 groups, as these differences were the largest. Out of 49 isolated tRNA genes with significantly different scores between TP0 and TP36, ∼53% had RPC4 peaks alone and 6% had RPC4 + H3K4me3 + RPB2 peaks, whereas among 82 non-changing Pol III-occupied isolated tRNA genes, these numbers were about 11% for genes with RPC4 peaks alone, and 52% for genes with RPC4 + H3K4me3 + RPB2 peaks (Supplementary Figure S8A). Thus, the majority of genes with changing scores, and those with the largest score differences between TP0 and TP36, were tRNA genes with RPC4 peaks alone, which tended to have lower scores at TP0 than genes in other groups (Figure 1). This was not a peculiarity of isolated tRNA genes, as a similar tendency was observed when all RPC4-occupied stable and changing tRNA genes were considered (Supplementary Figure S8B).

We used MEME to search for de novo motifs and CentriMo to search for known motifs within tRNA genes and their flanking sequences (±250 bp), or just within flanking sequences. We compared tRNA genes grouped according to surrounding peaks (tRNA genes in Figure 4A) as well as tRNA genes with or without significant RPC4 occupancy changes from TP0 to TP36 (tRNA genes in Figure 2B). We could not, however, identify any motifs differentially enriched in a specific group of tRNA genes, or in tRNA genes with changing versus stable RPC4 occupancy (data not shown), suggesting that differential expression after hepatectomy is not directly linked to the presence of specific DNA elements within tRNA genes and their immediate flanking sequences. Consistent with this observation, with the exception of some differences in the A and B boxes, the known promoter elements of tRNA genes, previous studies failed to identify *cis*-regulatory sequences correlating with differential tRNA gene expression in the adult liver (2), during development (20), or in proliferating versus diffenrentiated cells (34).

Last, we examined the chromosomal location of all of the 143 genes with significant increased RPC4 occupancy from TP0 to TP36. As shown in Figure 4C, the median of T statistics was highest for genes on chromosome 13, which, together with chromosomes 1 and 3, also seemed to harbor a disproportionate number of changing genes. We performed a chi-square independence test testing the null hypothesis that the distribution of changing versus stable Pol III genes on each chromosome was uniform. We obtained a *P*-value of 4.21e−12, indicating a strong association between chromosomal location and changes in Pol III occupancy. As shown in the mosaic plot in Supplementary Figure S9, chromosome 13 was indeed strongly enriched in changing genes, and depleted in stable genes, relative to expected values. Chromosomes 1 and 3 harbored a relatively high number of changing genes but contributed little to the total chi-square score.

tRNA genes in clusters and contact domains have been observed to share similar transcriptional dynamics during differentiation, as determined by comparing transcription changes at tRNA genes with the median fold change across tRNA gene clusters and domains to which they belong (5). Chromosome 13 contains a number of clustered tRNA genes. To explore how these and other tRNA gene clusters behaved, we first defined tRNA clusters as regions containing three or more tRNA genes, each spaced less than 5 kb from the next one, which gave a total of 42 clusters (listed in Supplementary Table S7). For each chromosome, we performed Kolmogorov-Smirnov tests to compare the distribution of RPC4 fold change among genes outside and within clusters, testing the null hypothesis that the two distributions are similar. With this stringent criterion, some clusters did indeed display a significantly different distribution of fold changes than non-clustered genes on the same chromosome, as shown by their *P*-values (Figure 4D (red bars indicate *P*-values < 0.05), Supplementary Table S7), but many did not. Thus, in our system, and as defined with stringent criteria, the fold changes for genes in clusters are not systematically differently distributed than for non-clustered genes on the same chromosome.

Supplementary Figure S10 shows examples of clusters with significantly different RPC4 fold change distribution from non-clustered genes on chromosomes 13 and 1 (see Figure 4D). The clusters contain very high proportions of changing Pol III loci and are strikingly devoid of nearby RPB2 peaks and, in many cases, of nearby H3K4me3 peaks, consistent with the results above indicating that the most changing tRNA genes are those devoid of RPB2 peaks (Figure 4A, Supplementary Figures S8A and B).

The Pol III occupancy changes described here contrast with the observation that during differentiation in human THP-1 cells, the most occupied genes were the ones with the biggest occupancy changes after differentiation (5). They are, however, consistent with previous results comparing Pol III occupancy in serum-replete versus serum-starved IMR90Tert human cells, which identified highly and relatively stably occupied Pol III genes, and Pol III genes with lower but more dynamic Pol III occupancy (4). It is furthermore striking that many of the genes with scores changing between TP0 and TP36 also changed scores between embryonic E15.5 and adult mouse liver (33). In the PH system, we further observe that stable, but not changing, genes, are close to H3K4me3 peaks and Pol II-occupied TSSs. It thus appears that cells have a network of Pol III genes with relatively stable expression, and genes outside this network to adapt Pol III transcription quickly and reversibly to the increasing need of dividing cells for Pol III products.

## DATA AVAILABILITY

The ChIP-seq data of this study have been submitted to he NCBI Gene Expression Omnibus (GEO; https://www.ncbi.nlm.nih.gov/geo/) under accession number GSE114650. UCSC Genome Browser session.

## Supplementary Material

Supplementary Data

## CycliX consortium

Nouria Hernandez^1^, Mauro Delorenzi^2,3,4^, Bart Deplancke^5^, Béatrice Desvergne^1^, Nicolas Guex^6^, Winship Herr^1^, Felix Naef^5^, Jacques Rougemont^7^, Ueli Schibler^8^, Teemu Andersin^8^, Pascal Cousin^1^, Federica Gilardi^1^, Pascal Gos^8^, Fabienne Lammers^1^, Maykel Lopes^1^, François Mange^1^, Shilpi Minocha^1^, Sunil Raghav^5^, Dominic Villeneuve^1^, Roberto Fabbretti^6^, Volker Vlegel^6^, Ioannis Xenarios^1,2,6^, Eugenia Migliavacca^1,6^, Viviane Praz^1,2^, Fabrice David^2,7^, Yohan Jarosz^2,7^, Dmitry Kuznetsov^6^, Robin Liechti^6^, Olivier Martin^6^, Julien Delafontaine^2,7^, Julia Cajan^5^, Cristian Carmeli^1,3^, Kyle Gustafson^1^, Irina Krier^5^, Marion Leleu^2,7^, Nacho Molina^5^, Aurélien Naldi^7^, Leonor Rib^1^, Jonathan Sobel^5^, Laura Symul^5^, Gergana Bounova^1,2^, Philippe Jacquet^2,7^

^1^Center for Integrative Genomics, Faculty of Biology and Medicine, University of Lausanne, 1015 Lausanne, Switzerland

^2^Swiss Institute of Bioinformatics, 1015 Lausanne, Switzerland

^3^Bioinformatics Core Facility, Swiss Institute of Bioinformatics, 1015 Lausanne, Switzerland

^4^Department of Oncology and Ludwig Center for Cancer Research, Faculty of Biology and Medicine, University of Lausanne, 1011 Lausanne, Switzerland

^5^Interfaculty Institute of Bioengineering, School of Life Sciences, Ecole Polytechnique Fédérale de Lausanne, 1015 Lausanne, Switzerland

^6^Vital IT, Swiss Institute of Bioinformatics, 1015 Lausanne, Switzerland

^7^Bioinformatics and Biostatistics Core Facility, School of Life Sciences, Ecole Polytechnique Fédérale de Lausanne, 1015 Lausanne, Switzerland

^8^Department of Molecular Biology, Faculty of Sciences, University of Geneva, 1211 Geneva, Switzerland
