# Supplementary material for: Differential regulation of RNA polymerase III genes during liver regeneration
Source: Nucleic Acids Res. 2018 Dec 29;47(4):1786–96. doi: 10.1093/nar/gky1282 (PMC6393285; doi:10.1093/nar/gky1282)
Supplement: Supplementary Data [file gky1282_supplemental_files.zip › Supplementary_Data.pdf]

Differential regulation of RNA polymerase III genes during liver regeneration

Meghdad Yeganeh†, Viviane Praz†, Cristian Carmeli†, Dominic Villeneuve, Leonor Rib, Nicolas Guex, Winship Herr, Mauro Delorenzi, Nouria Hernandez*, and the CycliX consortium

† First co-authors

*Corresponding author

Supplemental Inventory

Supplementary Figures

Figure S1.

Figure S2.

Figure S3.

Figure S4. Related to Figure 1

Figure S5. Related to Figure 1

Figure S6.

Figure S7.

Figure S8. Related to Figure 4A

Figure S9. Related to Figure 4C

Figure S10. Related to Figure 4D

Supplementary Tables (Separate files)

Table S1.

Table S2. Related to Figure 2C, Figure 2D, and Figure 2E.

Table S3. Related to Figure 1

Table S4. Related to Figure 2B and Figure 3D

Table S5. Related to Figure 3C, Figure 3E, and Figure 3F

Table S6. Related to Figure 4A and Figure 4B

Table S7. Related to Figure 4D

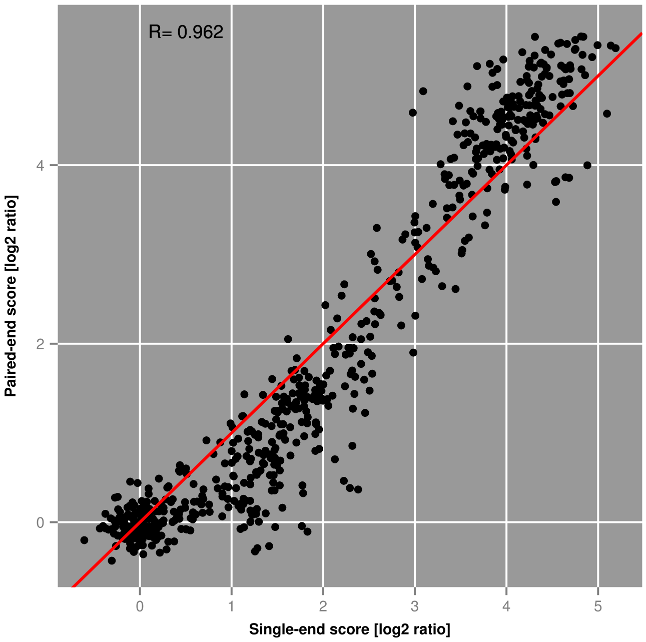

Figure S1. Comparison of scores obtained with paired-end sequencing (current study) and single-end sequencing (Renaud et al., Genome Res, 2014. 24(1): p. 37-51). The scores are log2 ratios with a pseudocount value of 30. The scores are the average of two biological replicates (mouse liver) for the single-end sequencing, and the average of two replicates at TP0 for the paired-end sequencing. Single-end and paired scores were normalized to the median number of counts of the four samples. The Pearson correlation coefficient of the two sets of scores is 0.962. Each dot represents a gene, the red line is the x=y line.

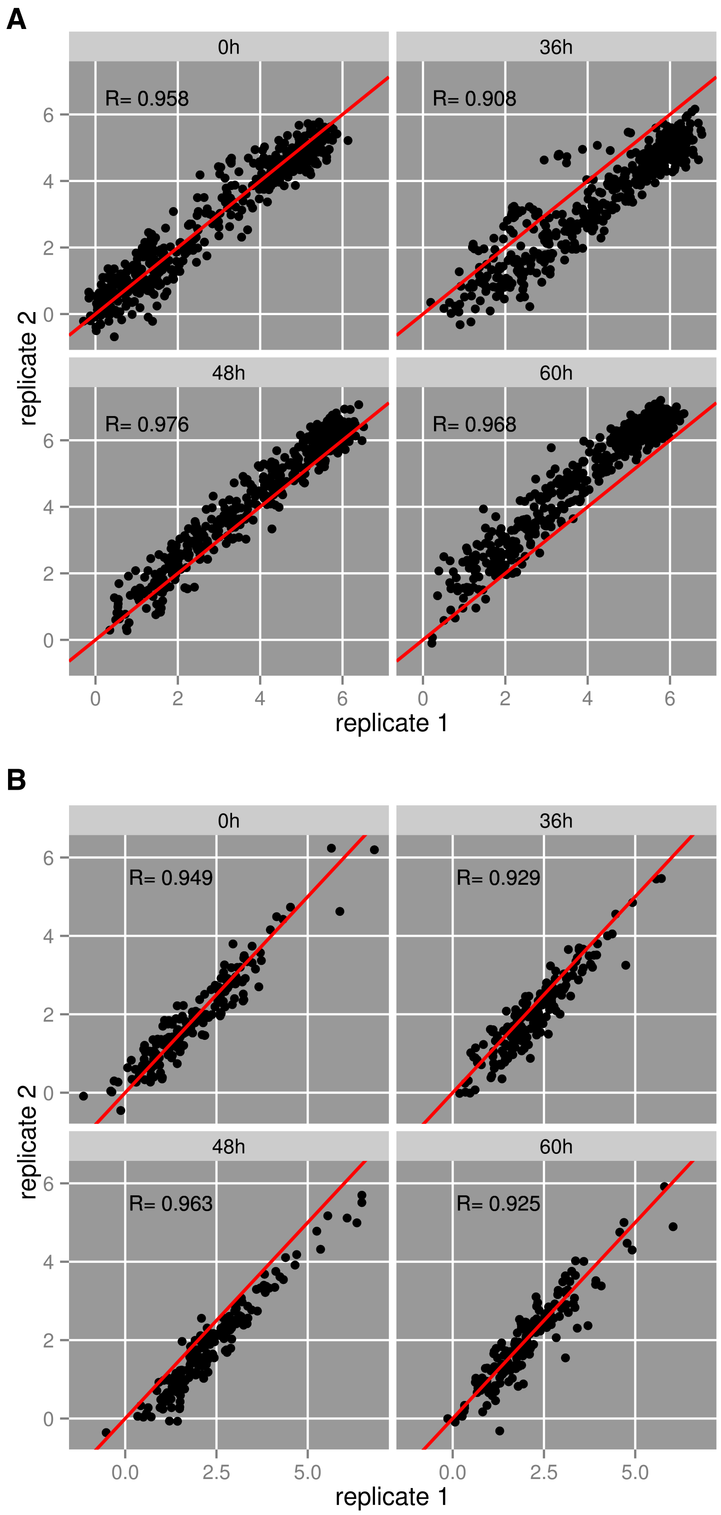

Figure S2. Scatterplot of RPC4 and RPB2 scores between the two replicates for each time point. The x=y line is in red. R is the Pearson correlation coefficient. **A)** RPC4 scores for those loci whose score is higher than the corresponding cutoff value for at least one time point. **B)** RPB2 scores for loci whose score is higher than the corresponding cutoff for at least one time point.

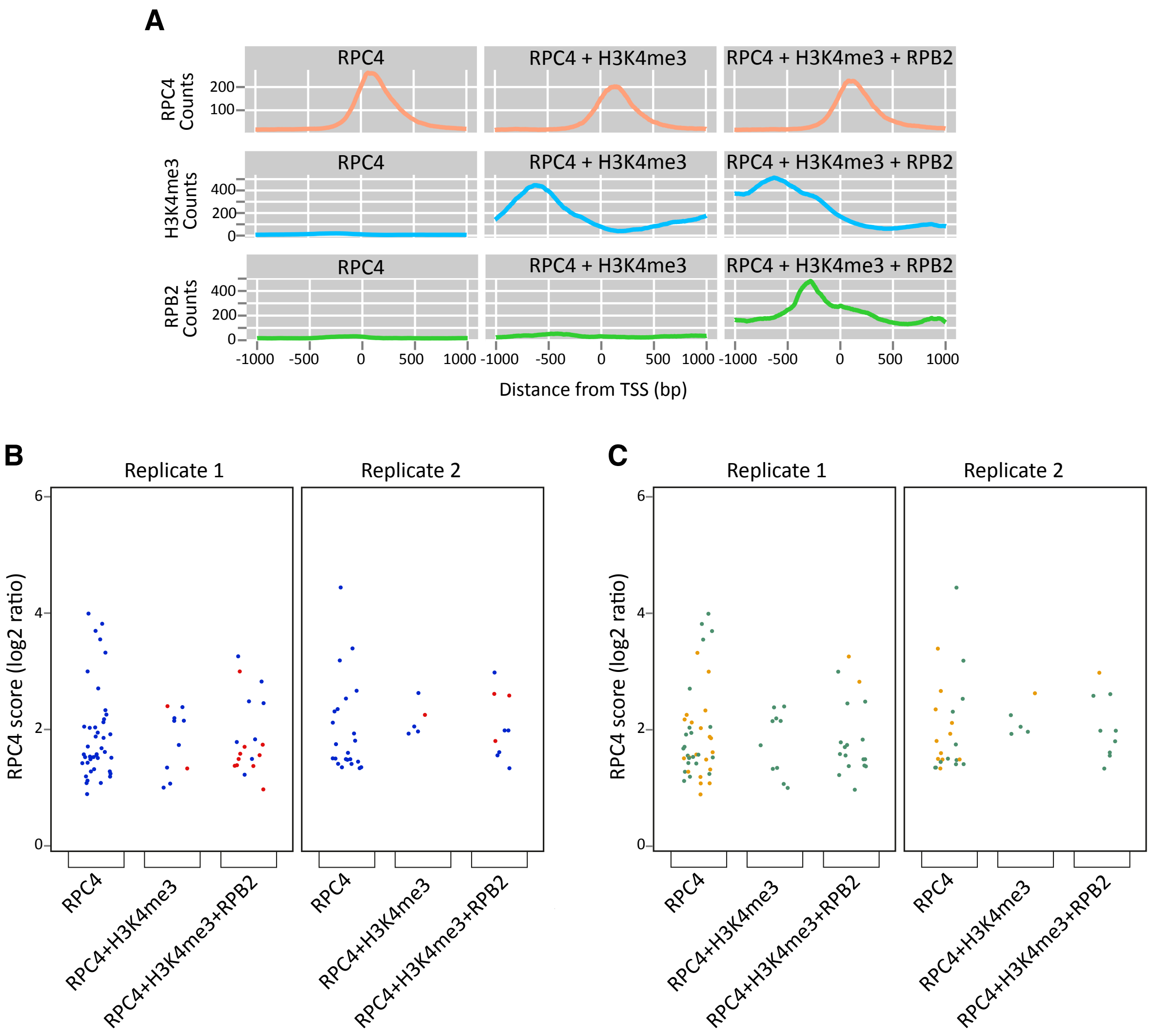

Figure S3. **A)** Average tag density profiles for RPC4, H3K4me3, and RPB2, as indicated on the left, for isolated SINEs with RPC4 peaks only (left column), RPC4 and H3K4me3 peaks (middle column), and RPC4, H3K4me3, and RPB2 peaks (right column). The profiles were computed from replicate 2 in a region of +/- 1Kbp around the annotated TSSs of isolated SINEs. **B)** Scatterplots of RPC4 scores for isolated SINEs in replicates 1 and 2 as indicated. The SINE groups are as in A, and the grouping into groups is done for each replicate independently (for H3K4me3, only replicate 2 was used). Scores in different groups are similar, permutation based t test P-value > 0.29) Red and blue dots, SINEs with and without associated CpG islands, respectively. **C)** As in B, but the orange and green dots indicate SINEs >2.65 Kbp and <2.65 Kbp, respectively, from TSS or poly A sites of Pol II genes.

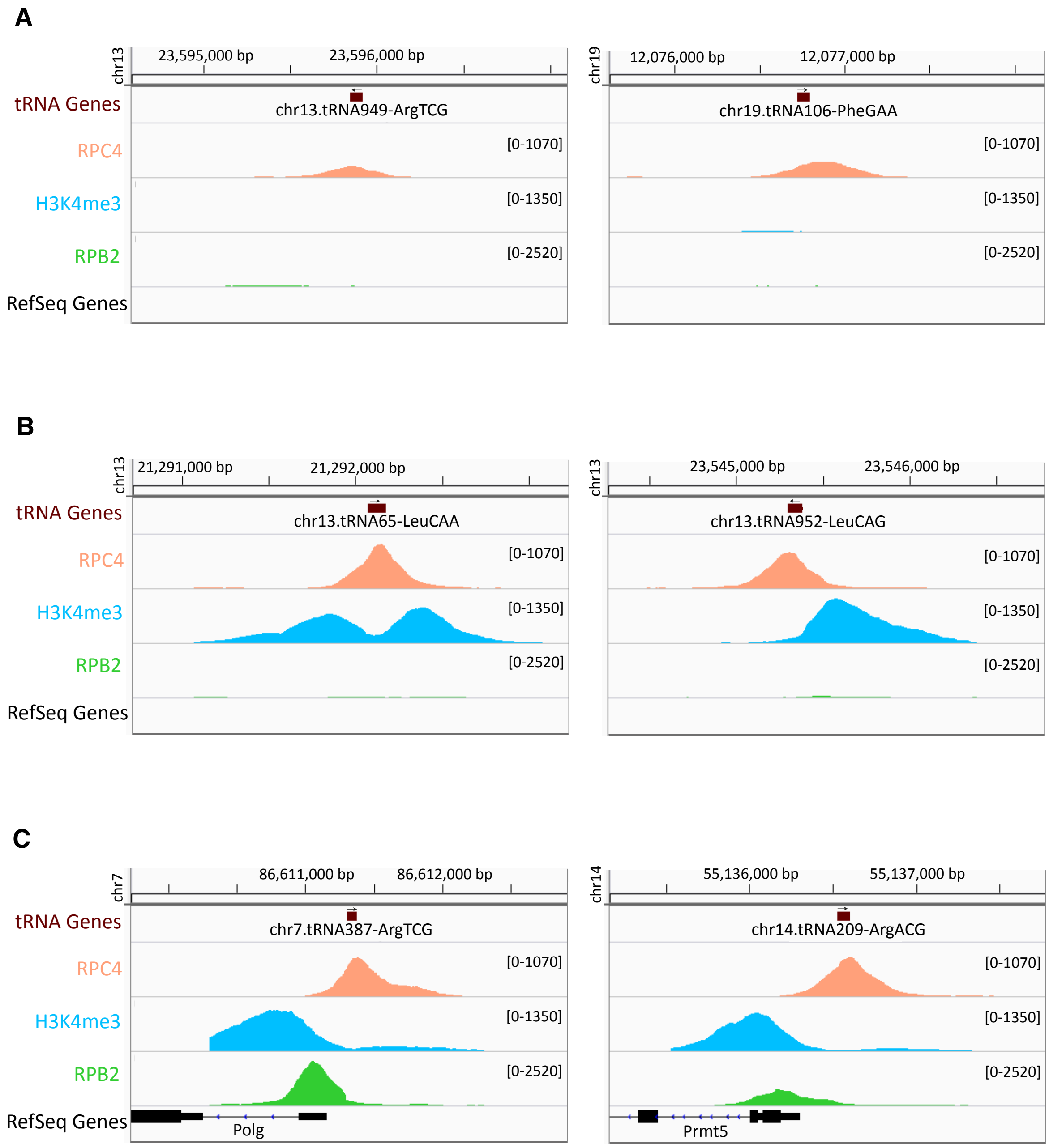

Figure S4. Integrative Genomics Viewer (IGV) views of genome regions surrounding isolated tRNA genes with: **A)** RPC4 peaks only. **B)** RPC4 and H3K4me3 peaks. **C)** RPC4, H3K4me3, and RPB2 peaks. The ChIP-seq tracks are from replicate 2.

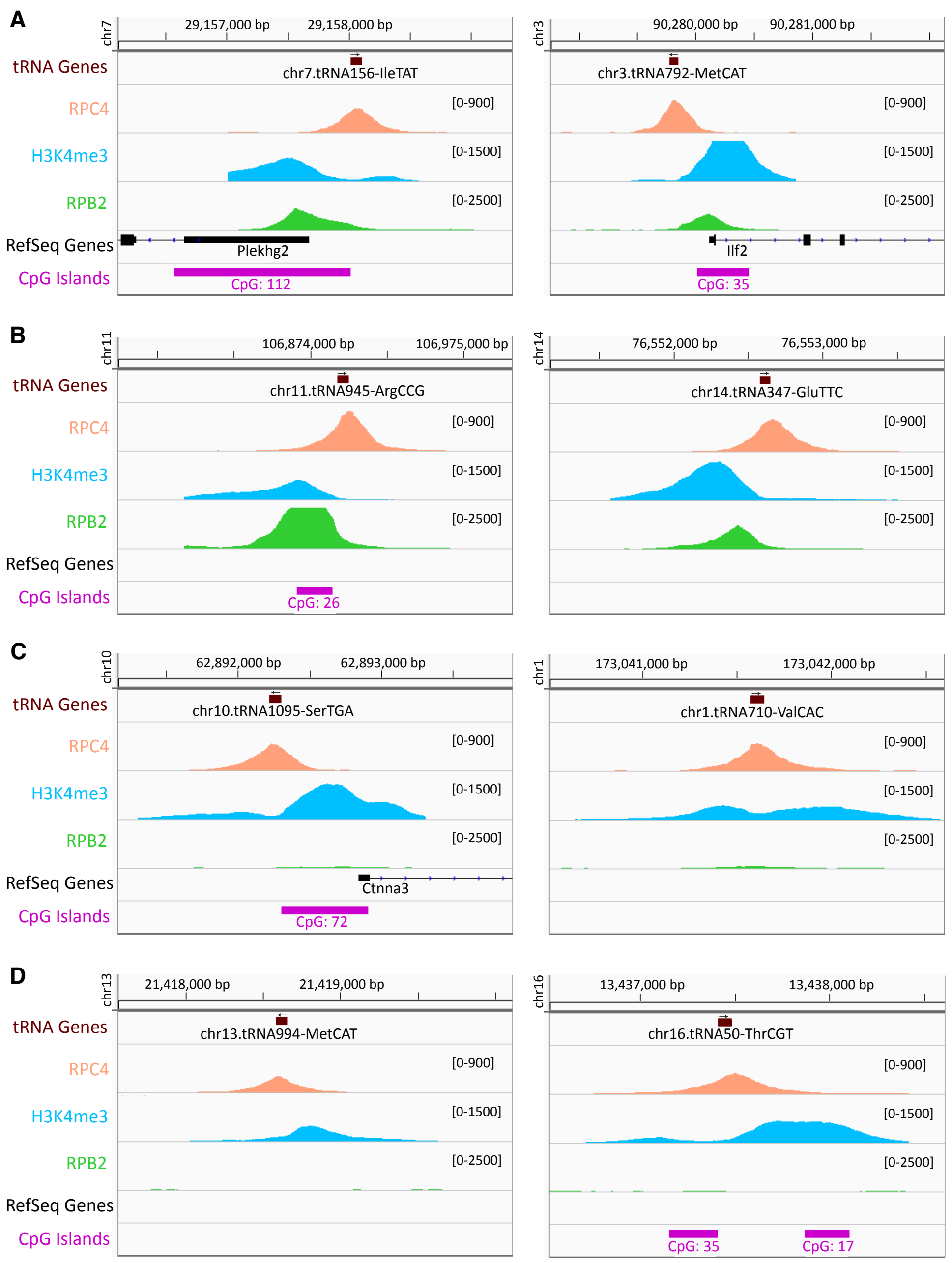

Figure S5. IGV views of genome regions surrounding isolated tRNA genes with different features: **A)** with RPC4, H3K4me3, and RPB2 peaks, located <2.65 Kbp from a Pol II gene, and with the RPB2 peak located on the Pol II gene TSS. **B)** with RPC4, H3K4me3, and RPB2 peaks, located >2.65 Kbp from a Pol II gene, and either containing (left), or not containing (right), a CpG island. **C)** with RPC4 and H3K4me3 peaks, and no RPB2 peak, located <2.65 Kbp from a Pol II gene, and with the main H3K4me3 peak either coinciding (left), or not coinciding (right), with a Pol II TSS and CpG island. **D)** with RPC4 and H3K4me3 peaks, and no RPB2 peak, located >2.65 Kbp from a Pol II gene. The ChIP-seq tracks are from replicate 2.

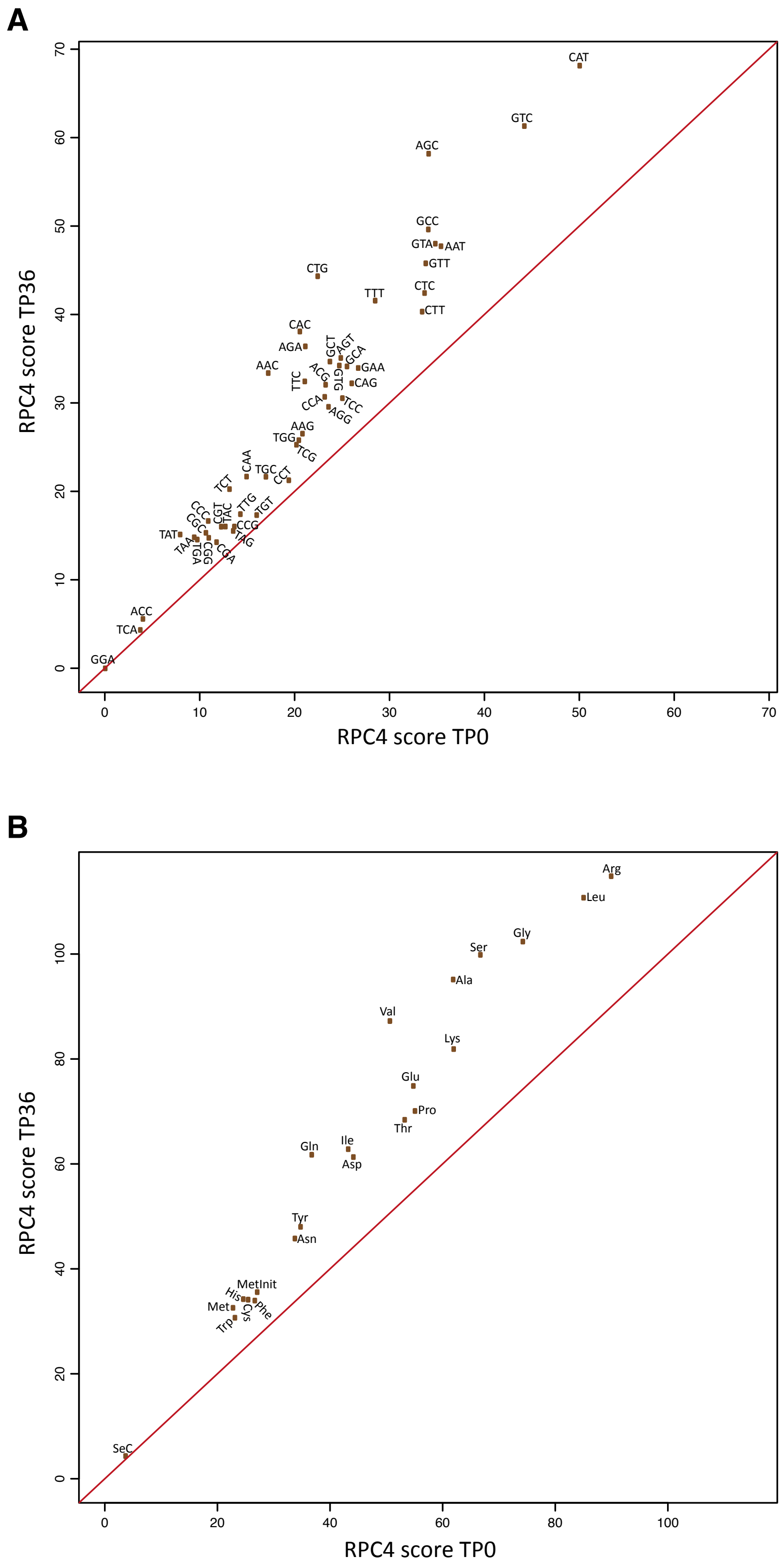

Figure S6. Scatter plots of RPC4 scores at TP0 and TP36 for all tRNA genes by **A)** Isoacceptors **B)** Isotypes. The scores were calculated as the mean of the two replicates for each gene, and then summed by isoacceptors or isotypes.

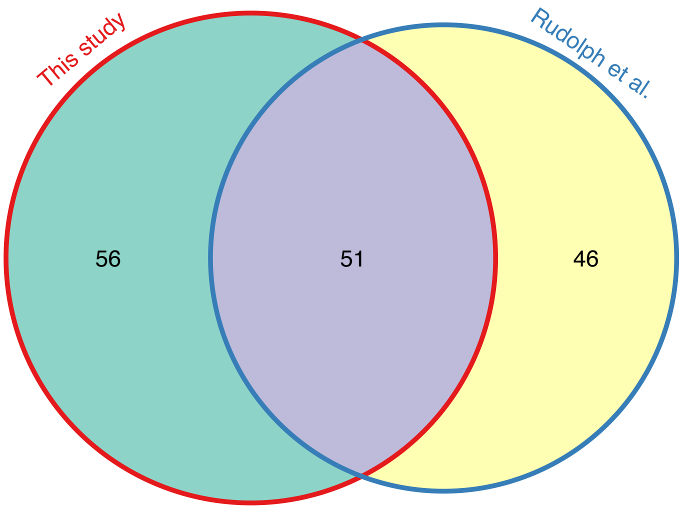

Figure S7. Venn diagram comparing tRNA genes differentially occupied by Pol III at TP0 and TP36 in this study with those different between embryonic and adult mouse liver (Rudolph et al., PLoS Genet, 2016. 12(5): p. e1006024). The number of active tRNA genes was 295 in our study and 311 in Rudolph et al.

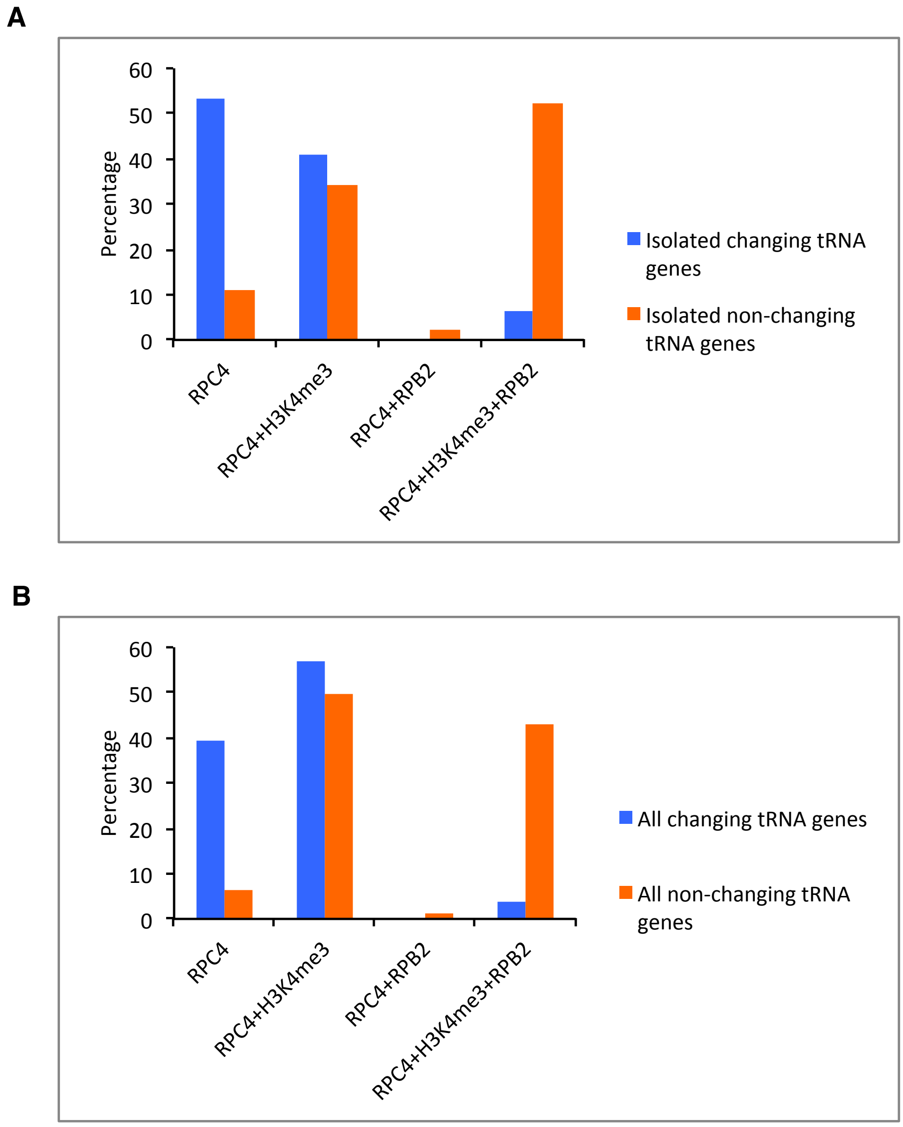

Figure S8. Percentage of tRNA genes in each group according to presence of different peaks, in changing and non-changing tRNA genes (with RPC4 score above cutoff) for: **A)** Isolated tRNA genes **B)** All (isolated and non-isolated) tRNA genes.

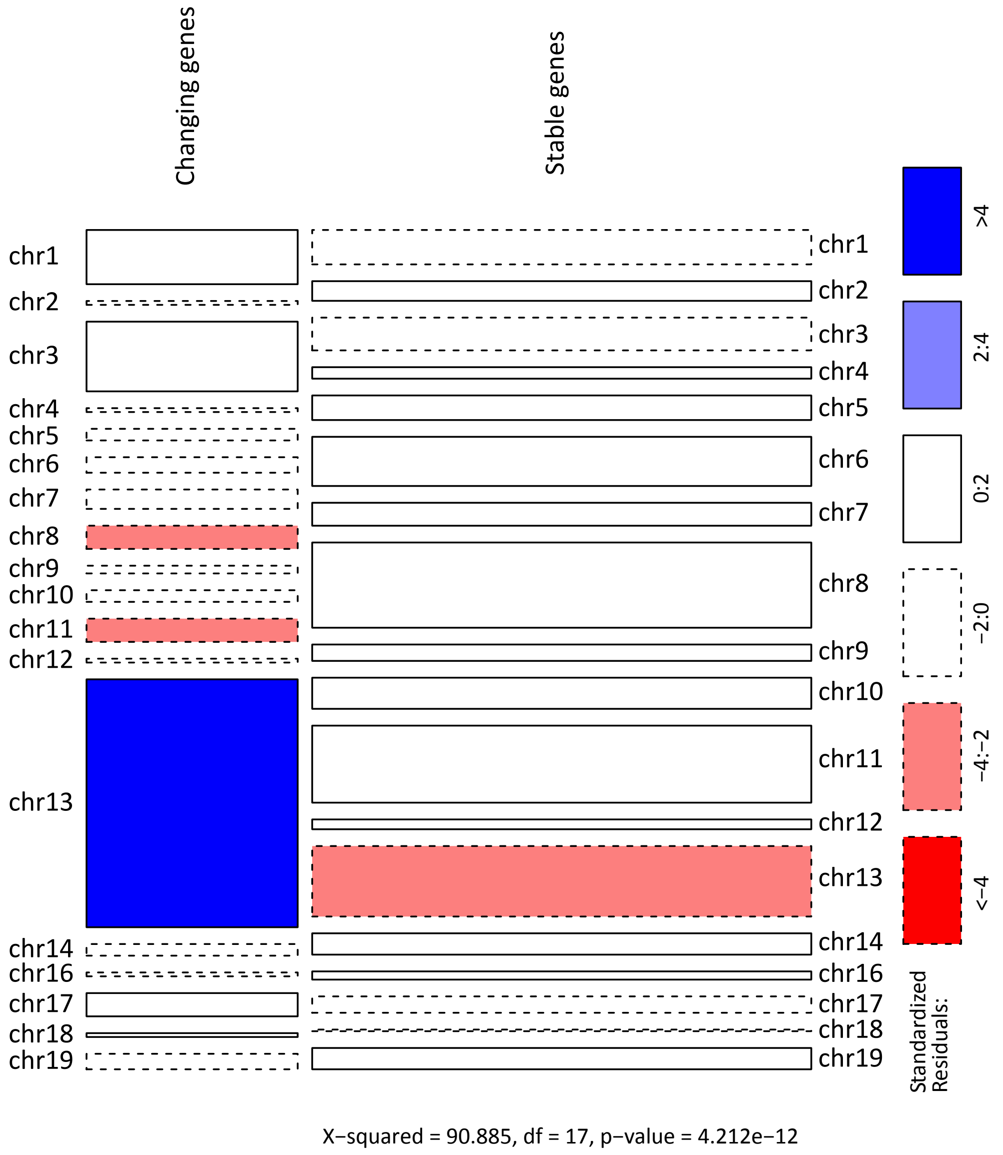

Figure S9. Chromosome 13 is enriched in Pol III genes with changes in occupancy between TP00 and TP36. Mosaic plot where the width of the two columns is related to the number of genes with significant (left column) or non-significant (right column) changes in Pol III occupancy scores between TP00 and TP36 relative to the total number of Pol III genes. The height of each rectangle in the first and second column is related to the number of changing or stable genes in each chromosome relative to the total number of changing or stable genes. For each cell, full or dashed lines indicate whether the relative contribution of each chromosome to the total Chi-square score is positive or negative, respectively, with blue and red colors indicating that the observed values are higher and lower, respectively, than expected (random) values.

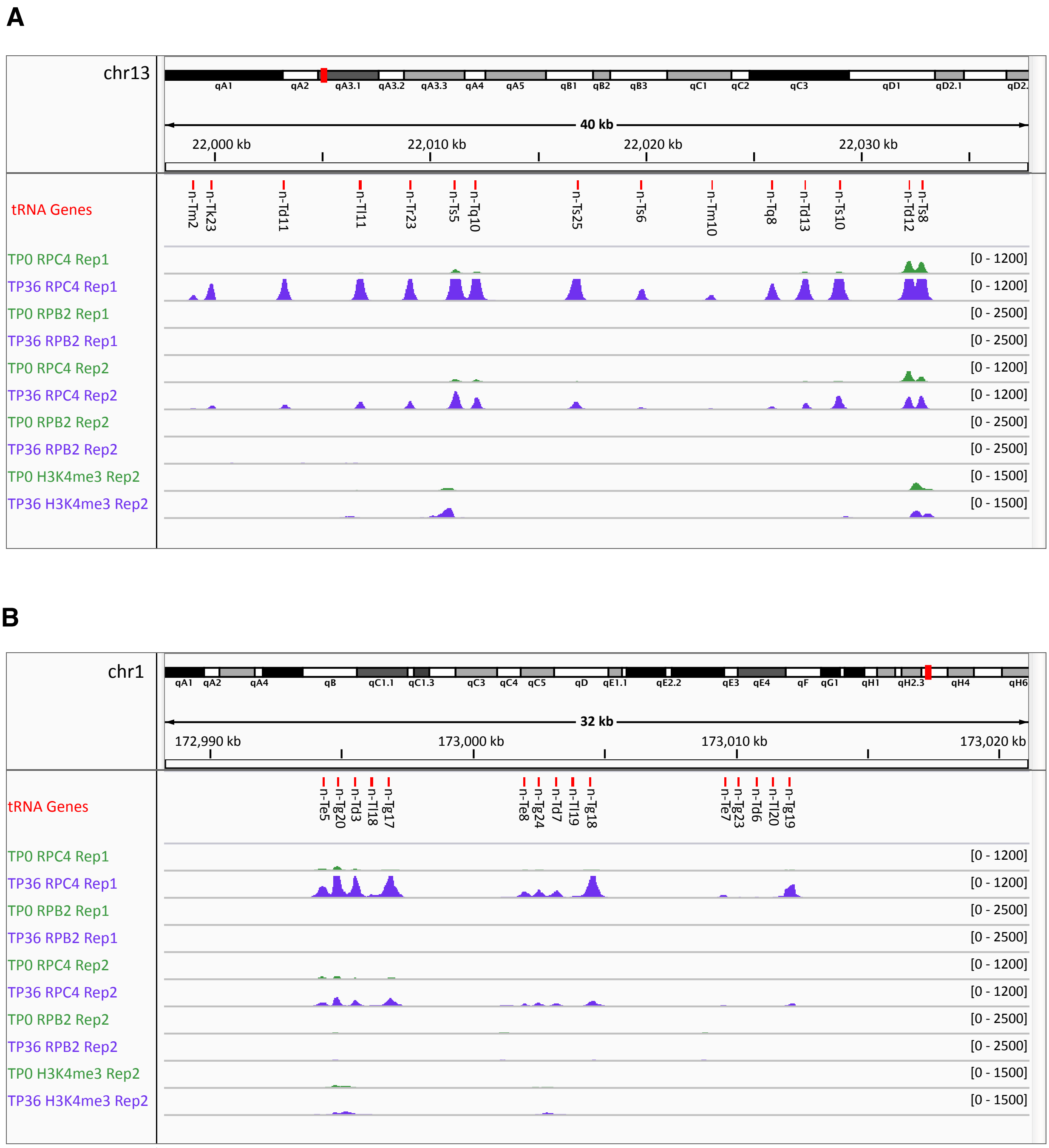

Figure S10. IGV views of genome regions corresponding to some clusters of tRNA genes on chromosomes 13 (cluster 172) and 1 (clusters 10,11,12). The tracks are as indicated on the left.
