# Supplementary material for: Differential regulation of RNA polymerase III genes during liver regeneration
Source: Nucleic Acids Res. 2018 Dec 29;47(4):1786–96. doi: 10.1093/nar/gky1282 (PMC6393285; doi:10.1093/nar/gky1282)
Supplement: Supplementary Data [file gky1282_supplemental_files.zip › Supplementary_Table_S2.docx]

| **Oligonucleotide name** | **Forward primer (5’ to 3’)** | **Reverse primer (5’ to 3’)** |
| --- | --- | --- |
| Pre-985 tRNA Arg TCT | GCATTGGACTTCTAGCATGATT | ACTCTGGCGGGACTCGAA |
| Pre-85 tRNA Leu CAA | CGTTCGCTTCCTCTACTGAG | TGTCAGAAGTGGGATTCGAA |
| Pre-960 tRNA Tyr GTA | CCTTCGATAGCTCAGTTGGT | CGACCTAAGGATATCAACACC |
| Tbp | AACAGCCTTCCACCTTATGC | CCGTAAGGCATCATTGGACT |
| Bdp1 | CAGAGTCTTGCATGGGACAA | TTGGCTCACTGTGTTCCTTC |
| Snapc1 | AGTCGTTACACCTGAGTATGC | AGCATTTTCTCTTCCTCTTGGG |
| Polr3h | GTGCACGTCTCTTTAGGGTT | CGCTTCATCAAACTTGGCAG |
| Polr3d | AGAAGGAAAAGCGTGAACGG | GGACCCTGCTCGAAGATAGA |
| Polr3c | GCCCTTTGTGTCCTCATTCA | CTTCATACTCCACCACACCG |
| Polr3f | TGACTATCATTGCTGCGAAGG | TAGGTGGGAGGATTGGGTTG |
| Polr3e | AGAGACAGTTTGTGCTCACG | TGAGACGCCACTGAAGAGTA |
| Actb | CTAAGGCCAACCGTGAAAAGAT | CACAGCCTGGATGGCTACGT |
| Polr3g | GATGTTGTGTTGAAGCCTCC | AAGGCCAGCATGTAATCCTC |
| Polr3gl | TCGATTGGAACCCTGATTGG | CCGCTCCTTCTGTACTTTCC |

**Table S2.** Sequences of oligonucleotides used for qPCR experiments. This table is related to Figure 2C, Figure 2D, and Figure 2E.
